# LncRNA BBOX1‐AS1 upregulates HOXC6 expression through miR‐361‐3p and HuR to drive cervical cancer progression

**DOI:** 10.1111/cpr.12823

**Published:** 2020-06-09

**Authors:** Jun Xu, Baohua Yang, Lifeng Wang, Yunheng Zhu, Xiuxiang Zhu, Ziyin Xia, Zhen Zhao, Ling Xu

**Affiliations:** ^1^ Department of Obstetrics and Gynecology Minhang Hospital Fudan University Shanghai China; ^2^ Department of Clinical Laboratory Minhang Hospital Fudan University Shanghai China

**Keywords:** BBOX1‐AS1, CC, HOXC6, HuR, miR‐361‐3p

## Abstract

**Objectives:**

Over the past years, growing attention has been paid to deciphering the pivotal role of long non‐coding RNAs (lncRNAs) in regulating the occurrence and development of human malignancies, cervical cancer (CC) included. Nonetheless, the regulatory role of lncRNA BBOX1 antisense RNA 1 (BBOX1‐AS1) has not been explored as yet.

**Material and Methods:**

The expression of BBOX1‐AS1 was detected by reverse transcription real‐time quantitative polymerase chain reaction (RT‐qPCR). Cell Counting Kit‐8 (CCK‐8), colony formation, TUNEL, Western blot, transwell and immunofluorescence assays testified the critical role of BBOX1‐AS1 in CC. The relationship between RNAs (BBOX1‐AS1, miR‐361‐3p, HOXC6 and HuR) was analysed by luciferase reporter, RNA Immunoprecipitation (RIP) and RNA pull‐down assays.

**Results:**

BBOX1 antisense RNA 1 antisense RNA 1 was revealed to be highly expressed in CC. Decreased expression of BBOX1‐AS1 had suppressive effects on CC cell growth and migration. Molecular mechanism assays verified that BBOX1‐AS1 had negative interaction with miR‐361‐3p in CC. Additionally, homeobox C6 (HOXC6) was validated to be a downstream target of miR‐361‐3p in CC. Furthermore, ELAV‐like RNA‐binding protein 1, also known as HuR, was uncovered to be capable of regulating the mRNA stability of HOXC6 in CC. More importantly, rescue assays delineated that knockdown of HuR after overexpressing miR‐361‐3p could reverse BBOX1‐AS1 upregulation‐mediated effect on CC progression. Similarly, the function induced by BBOX1‐AS1 upregulation on CC progression could be countervailed by HOXC6 depletion.

**Conclusions:**

BBOX1 antisense RNA 1 facilitates CC progression by upregulating HOXC6 expression via miR‐361‐3p and HuR.

## 1 INTRODUCTION

Recognized as a type of fatal malignancy belonging to gynaecological tumours, cervical cancer (CC) has been diagnosed as a part of the dominating cause of cancer‐linked mortality in women worldwide.^1^, ^2^ Besides, previous reports have revealed that CC is one of the primary factors that give rise to cancer‐related death cases in China, rural region in particular. Despite the application of human papillomavirus vaccine in the past years, the mortality of CC patients remains a 10‐fold rise in developed countries than that in developing countries, which indicates that CC remains a major stumbling block for human health.^3^ Most patients with early CC might be cured by surgical operation.^4^ Nonetheless, with regard to those CC patients at advanced stages, there are no or limited effective treatments and thus the survival rate of these patients remains low.^5^ Radical hysterectomy, pelvic lymphadenectomy, radiotherapy together with chemotherapy are the main therapeutic methods of CC in recent years.^6^ For the purpose of making further progress in CC treatment, researchers have been dedicated to identifying and developing biomarkers, which are capable of predicting CC biological behaviours.^7^ Given that the crucial biological courses involved in CC are extremely complicated, it is urgently needed to further decipher the molecular mechanisms in CC.

Non‐coding RNAs (ncRNAs) are commonly accepted as the potential key factors in gene regulation, eliciting impact on non‐tumour and tumour cell phenotypes.^8^, ^9^ Identified as a subtype of ncRNAs, long non‐coding RNAs (lncRNAs) are generally considered to be genomic transcriptions with a length of exceeding 200 nucleotides and have no or limited protein‐coding capacity because of lacking an open reading structure of a necessary length.^10^, ^11^ Multiple studies have associated lncRNAs with the molecular mechanisms underlying cancer progression and emphasized their significance as promising biomarkers and therapeutic targets.^12^ Previous investigations have elucidated the critical effect of lncRNAs on the regulation of a string of complex biological behaviours, such as gene expression, cellular growth as well as transcription.^13^, ^14^ More importantly, abnormally expressed lncRNAs have been implicated in the initiation and progression of diverse cancers by functioning as a competing endogenous RNA (ceRNA). For example, lncRNA EPB41L4A‐AS2 restrains hepatocellular carcinoma progression via miR‐301a‐5p/FOXL1 axis.^15^ LncRNA NORAD promotes CC progression by sponging miR‐590‐3p to upregulate SIP1 expression.^16^ Besides, existing researches have also revealed that lncRNAs can regulate tumour progression through RNA‐binding proteins (RBPs).^17^ BBOX1 antisense RNA 1 (BBOX1‐AS1) is a novel lncRNA in CC research. Its regulatory roles in CC are unclear and thus worth exploring.

In the present study, we attempted to decipher the regulatory function of BBOX1‐AS1 on CC cell proliferation, apoptosis as well as metastasis. Data from this study validate that BBOX1‐AS1 upregulates homeobox C6 (HOXC6) expression through miR‐361‐3p and HuR to facilitate CC progression, with implications of BBOX1‐AS1 as a potential biomarker for CC treatments.

## 2 MATERIALS AND METHODS

### 2.1 Tissue samples

100 CC tissues and the corresponding non‐tumour tissues were collected from Minhang Hospital, Fudan University, between 2013 and 2018. All patients did not receive any therapy before surgery. Fresh samples were maintained in liquid nitrogen and preserved at −80°C. Informed consents were signed by every participant, with the approval obtained from the Ethics Committee of Minhang Hospital, Fudan University.

### 2.2 Microarray analysis

TRIzol reagent (Invitrogen) was employed for extracting total RNAs from 3 CC tissues and matched normal samples. Different expressions of lncRNAs were acquired from whole‐genome microarray expression profiling with a cut‐off criteria of *P* < .05 and log2 > 2. The microarray analysis of differentially expressed lncRNAs was conducted, and R program was used for gathering differentially expressed genes.

### 2.3 Cell culture

Human normal cervical epithelial cell line (H8) and human CC cell lines (C33A, CaSki, HeLa, SiHa) were bought from Chinese Academy of Sciences. Cells were cultured in a moist incubator containing 5% CO_2_ at 37°C, with Dulbecco's Modification of Eagle's Medium (DMEM) (Invitrogen), which was added with 10% foetal bovine serum (FBS; Invitrogen) and 1% penicillin/streptomycin (Sigma‐Aldrich).

### 2.4 Cell transfection

SiHa and HeLa cells were transfected with the specific short hairpin RNAs to BBOX1‐AS1 (sh‐BBOX1‐AS1#1#2), HuR (sh‐HuR#1#2), HOXC6 (sh‐HOXC6#1#2) and the negative control (sh‐NC), as well as pcDNA3.1/BBOX1‐AS1, pcDNA3.1/HOXC6 and the empty pcDNA3.1 vector (all acquired from GenePharma), separately. The miR‐361‐3p mimics and NC mimics were simultaneously obtained from GenePharma. Lipofectamine 2000 (Invitrogen) was applied for cell transfection for 48 hours.

### 2.5 RT‐qPCR

Total RNA was extracted by TRIzol Reagent (Invitrogen) and then reverse‐transcribed into cDNA using Reverse Transcription Kit (Takara). Next, real‐time quantitative polymerase chain reaction (RT‐qPCR) was carried out in Bio‐Rad CFX96 system utilizing SYBR Green Real‐Time PCR Kit (Takara). Fold expression changes were calculated with the use of 2^−∆∆Ct^ method, and GAPDH/U6 was regarded as an internal control.

### 2.6 Subcellular fractionation

Cytoplasmic and nuclear RNA from SiHa and HeLa cells were isolated by using the Cytoplasmic & Nuclear RNA Purification Kit (Norgen). The expression level of BBOX1‐AS1 was measured using RT‐qPCR, and GAPDH/U6 was cytoplasmic control or nuclear control.

### 2.7 FISH assay

The BBOX1‐AS1 FISH probe was synthesized by RiboBio. Cells were placed on culture slides and fixed with 4% paraformaldehyde (Sigma‐Aldrich), and then were blocked with pre‐hybridization buffer for 4 hours. FISH probe was added into the hybridization mixture overnight. Afterwards, slides were cleaned in washing buffer containing Saline sodium citrate. 4',6‐diamidino‐2‐phenylindole (DAPI) stained cells later. Lastly, cells were surveyed via a fluorescence microscope (Olympus).

### 2.8 CCK‐8 assay

1 × 10^4^ SiHa or HeLa cells were inoculated on fresh 96‐well plates and cultured over specific time points. Cell Counting Kit‐8 (CCK‐8) solution was then added for incubating for another 4 hours. Cell viability was determined by the utilization of a microplate reader to examine the absorbance at 450 nm.

### 2.9 Colony formation assay

Cells (5 × 10^3^) were incubated in 6‐well plates. After 2 weeks of incubation, colonies were fixed in of 4% paraformaldehyde for 30 minutes and were dyed in crystal violet (Sigma‐Aldrich) for 15 minutes, respectively. Visible colonies were counted manually via a microscope (Olympus).

### 2.10 TUNEL assay

Cell apoptosis was examined through TUNEL staining assay using the In Situ Cell Death Detection Kit (Roche). SiHa and HeLa cells were dyed with utilization of 4',6‐diamidino‐2‐phenylindole (DAPI) (Haoran Biotechnology) or Merge (Gene Denovo), severally. Relative fluorescence intensity was detected via an EVOS FL microscope (Invitrogen).

### 2.11 Western blot

The extraction of total protein was completed by using RIPA lysis buffer (Beyotime) containing protease inhibitors (Beyotime). Equivalent quantities of proteins were isolated by SDS‐PAGE, followed by moving to PVDF membrane (Millipore). The membranes were sealed with skimmed milk and then were incubated with primary antibodies for Bcl‐2 (ab185002), Bax (ab32503), cleaved‐caspase3 (ab2302), total‐caspase3 (ab197202), cleaved‐caspase6 (ab2326), total‐caspase6 (ab250847), cleaved‐caspase9 (ab2324), total‐caspase9 (ab219590), cleaved ‐PARP (ab32064), total‐PARP (ab74290), MMP2 (ab97779), MMP9 (ab219372), E‐cadherin (ab194982), N‐cadherin (ab202030), Vimentin (ab193555), Slug (ab51772), Twist (ab187008), HuR (ab200342), HOXC6 (ab151575), U2AF2 (ab37530), ELAV‐like RBP 1 (ELAVL1) (ab200342), LARP4B (ab197085), RBM10 (ab224149) and glyceraldehyde‐3‐phosphate dehydrogenase (GAPDH) (ab8245) from Abcam (Cambridge, USA). HRP‐conjugated secondary antibodies were added to incubate for 1 hour at dark room. GAPDH was an internal control. And the amount of protein was evaluated by chemiluminescence detection system.

### 2.12 Transwell assay

Cell migration was assessed by utilizing transwell chambers (Millipore). 2 × 10^4^ SiHa and HeLa cells were re‐suspended in serum‐free medium and added into top compartment, and medium containing 10% FBS was added to the bottom compartment. Cell invasion was analysed using Matrigel‐coated transwell chamber. After 48 hours, 4% paraformaldehyde and crystal violet were employed for fixation and coloration of cells. Finally, the number of migrating or invading cells was counted by an inverted microscope (Olympus).

### 2.13 RNA pull‐down assay

RNA pull‐down assay was run as requested by the guidelines of Pierce Magnetic RNA‐Protein Pull‐Down Kit (Thermo Fisher Scientific). Protein extracts from SiHa or HeLa were subjected to 50 pmol of biotinylated RNAs and M‐280 streptavidin magnetic beads (Invitrogen) at 4°C for 1 hour. The beads were collected via centrifugation, followed by RT‐qPCR or Western blot analysis.

### 2.14 Luciferase reporter assay

The wild‐type (WT) and mutant (Mut) binding sites of miR‐361‐3p to BBOX1‐AS1 were sub‐cloned into pmirGLO dual‐luciferase vector to construct BBOX1‐AS1‐WT and BBOX1‐AS1‐Mut. Then, plasmids were co‐transfected with miR‐361‐3p mimics, NC mimics or miR‐361‐3p mimics + pcDNA 3.1/HOXC6 into SiHa and HeLa cells. The luciferase activity was detected via Dual‐Luciferase Reporter Assay System (Promega).

### 2.15 RIP assay

RNA Immunoprecipitation (RIP) experiment was processed using Magna RIP™ RNA‐Binding Protein Immunoprecipitation Kit (Millipore) in line with the user guide. RIP lysis buffer was used for lysing SiHa and HeLa cells at 4°C for 30 minutes. Then, the cell extracts were incubated with magnetic beads conjugated to human anti‐Ago2 antibody (Millipore) or anti‐HuR antibody (Millipore). Normal mouse immunoglobulin G (IgG; Millipore) acted as the negative control. The relative RNA expression level was analysed using RT‐qPCR.

### 2.16 Actinomycin D assay

The transfected cells were treated with Actinomycin D at a concentration of 1‐5 μmol/L for specific time points prior to RNA extraction with TRIzol reagent. Later, changes in RNA levels were analysed via RT‐qPCR.

### 2.17 Immunofluorescence

Cells were fixed in paraformaldehyde and then permeabilized by utilizing 0.1% Triton X‐217100 for 30 minutes. After blocking with 5% BSA, the primary antibodies against E‐cadherin and N‐cadherin were added at 4°C overnight, followed by cultivating with secondary antibodies in PBS for 1 hour. Later, cells were rinsed and counterstained using DAPI (Sigma‐Aldrich). Cells were observed and analysed under a confocal microscope (Olympus).

### 2.18 Xenograft assay in mice

The male nude mice were purchased from Shi Laike Company for animal study, with the approval obtained from the Ethics Committee of Minhang Hospital, Fudan University. Cells transfected with sh‐BBOX1‐AS1#1 or sh‐NC were subcutaneously injected into the flanks of each nude mouse. The growth of tumours was recorded every 4 days. On the 4 weeks after inoculation, mice were killed and tumours were resected and weighed for subsequent in vivo analysis.

### 2.19 Immunohistochemistry assay

Fresh tissues from in vivo assay were fixed in paraformaldehyde at first. After that, the fixed specimens were dehydrated in ethanol solutions, inserted into paraffin and cut into 4 μm thickness. The sections were cultivated with primary antibodies against Ki67 (Santa Cruz Biotechnology), PCNA (Santa Cruz Biotechnology), E‐cadherin or N‐cadherin overnight at 4°C, followed by HRP‐conjugated secondary antibodies. All sections were then visualized by OLYMPUSBX‐41 microscope (Olympus).

### 2.20 Haematoxylin‐eosin staining

Xenograft tissue samples in each group were collected and fixed by paraformaldehyde. Following embedding by paraffin, the serial 4 μm sections were treated with conventional haematoxylin‐eosin staining.

### 2.21 Statistical analysis

SPSS 18.0 software package (Chicago, IL, USA) was used for analysis of statistics in this study. Experimental data were manifested as mean ± SD. Student's *t* test and one‐way ANOVA were utilized for the group difference analysis. Kaplan‐Meier method was responsible for analysis of the overall survival of CC patients in different groups. Pearson's correlation analysis was used for conducting gene correlation analysis. *P* < .05 was seen as statistically significant. All experiments were run thrice independently in requirements.

## 3 RESULTS

### 3.1 BBOX1‐AS1 expression is significantly elevated in CC tissues and cells

**FIGURE 1:**
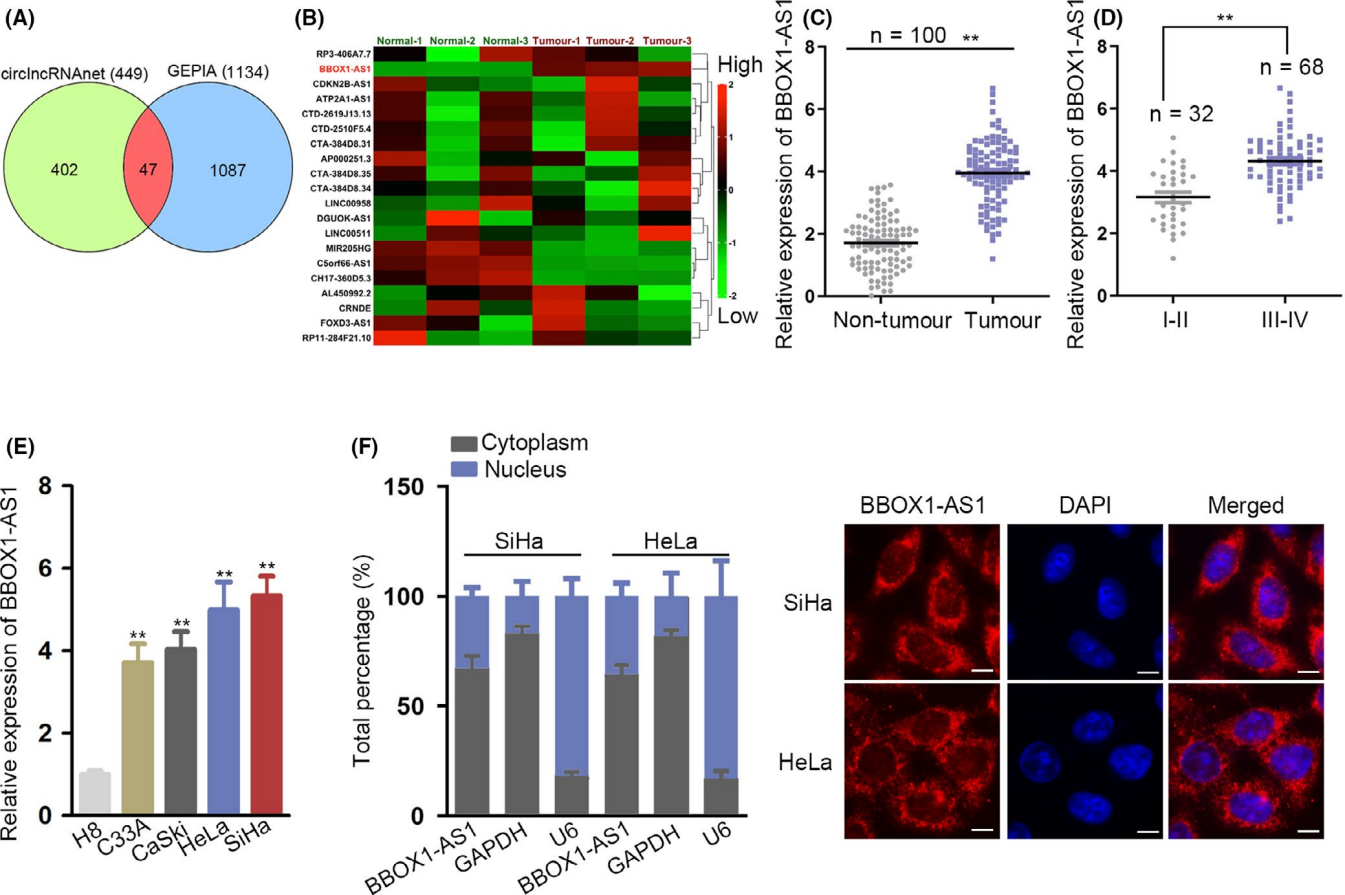
BBOX1 antisense RNA 1 (BBOX1‐AS1) expression is significantly elevated in cervical cancer (CC) tissues and cells. A, Venn diagram showed the overlaps of the analysis. B, The expression levels of these long non‐coding RNAs were detected by RT‐qPCR in 3 paired CC tissues and corresponding non‐cancer tissues. C, RT‐qPCR detected BBOX1‐AS1 expression in CC tissues and matched normal tissues. D, BBOX1‐AS1 expression was analysed via RT‐qPCR in early or advanced stages of CC patients. E, The expression of BBOX1‐AS1 in CC cell lines (C33A, CaSki, HeLa and SiHa) and normal cervical epithelial cell line (H8) was examined by RT‐qPCR. F, Subcellular fractionation and FISH assays were adopted for the detection of the subcellular localization of BBOX1‐AS1 in CC cells. ^**^ *P* < .01

To investigate the potential molecular mechanisms in CC, we first applied bioinformatics tools, circlncRNAnet (http://app.cgu.edu.tw/circlnc/) and GEPIA (http://gepia.cancer‐pku.cn/) to find the underlying lncRNAs that may play significant roles in regulation of CC progression. Venn diagram showed the overlaps of 449 lncRNAs with increased expression in CC tissues (log2FC > 1.5, *P* < .01) from circlncRNAnet and 1134 genes with increased expression in CC tissues (log2FC > 1.5, *P* < .01) from GEPIA, and then, 47 lncRNAs with high expression were obtained and listed (Figure 1A; Table S1). Subsequently, the expression levels of these lncRNAs were detected in 3 paired CC tissues and corresponding non‐cancer tissues. The result revealed the most significant upregulation of BBOX1‐AS1 in CC tissues (Figure 1B). Additionally, a notably elevated expression of BBOX1‐AS1 in CC tissues was uncovered through GEPIA (Figure S1A). Herein, we selected BBOX1‐AS1 to proceed with the study. Follow‐up RT‐qPCR analysis uncovered that compared with matched normal tissues, BBOX1‐AS1 expression was markedly upregulated in CC tissues (Figure 1C). Besides, a conspicuous rise of BBOX1‐AS1 expression was detected in advanced stages of CC patients (Figure 1D). In addition, through Kaplan‐Meier analysis, we observed that the overall survival rate of CC patients with high expression of BBOX1‐AS1 was lower than that with low expression of BBOX1‐AS1, suggesting that BBOX1‐AS1 upregulation was closely linked to poor prognosis (Figure S1B). Moreover, clinical data uncovered that BBOX1‐AS1 expression was tightly related to tumour size, differentiation, FIGO stage and distant metastasis (Table S2). Consistently, the expression of BBOX1‐AS1 was notably higher in CC cell lines (C33A, CaSki, HeLa and SiHa) than that in normal cervical epithelial cell line (H8) (Figure 1E). Furthermore, subcellular fractionation and FISH assays were adopted for the detection of the subcellular localization of BBOX1‐AS1 in CC cells. And the outcome revealed that BBOX1‐AS1 was mainly distributed in the cytoplasm of SiHa and HeLa cells (Figure 1F). Briefly, in tissues and cells of CC, BBOX1‐AS1 expression is evidently upregulated, and upregulation of BBOX1‐AS1 indicates poor prognosis of CC patients.

### 3.2 Low expression of BBOX1‐AS1 represses CC progression

**FIGURE 2:**
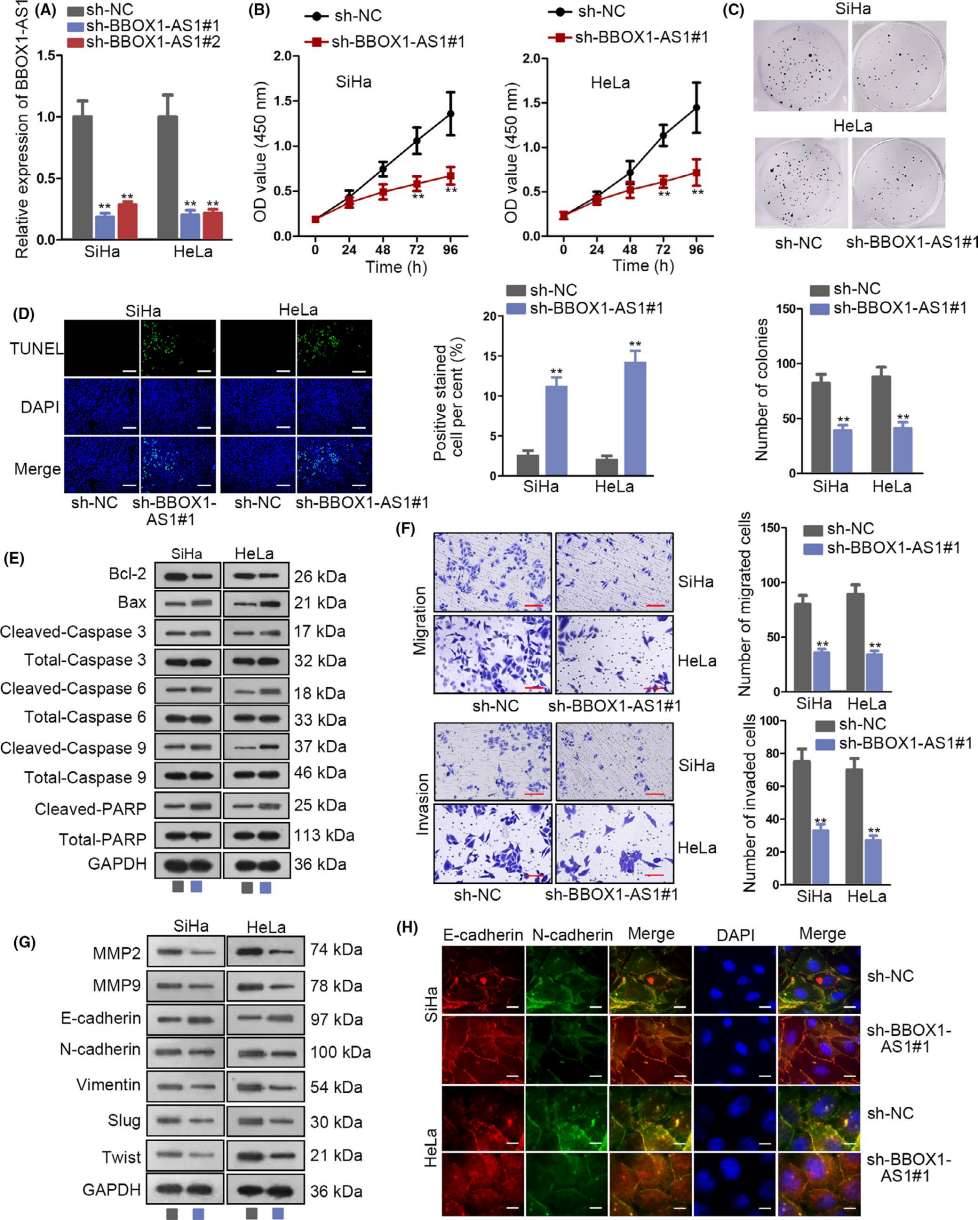
Low expression of BBOX1 antisense RNA 1 (BBOX1‐AS1) represses cervical cancer (CC) progression. A, The efficiency of BBOX1‐AS1 knockdown in SiHa and HeLa cells was measured by RT‐qPCR. B and C, The proliferation ability of SiHa and HeLa cells transfected with sh‐BBOX1‐AS1#1 or sh‐NC was evaluated by CCK‐8 and colony formation assays. D and E, TUNEL and Western blot were carried out to verify the promoting function of BBOX1‐AS1 knockdown on cell apoptosis. F‐H, Cell metastasis in transfected cells was analysed by transwell, Western blot and immunofluorescence. ^**^ *P* < .01

On the basis of the above findings, we aimed to study the biological function of BBOX1‐AS1 in CC by use of loss‐of‐function assay. In the first place, RT‐qPCR analysis unveiled that BBOX1‐AS1 expression was the lowest in sh‐BBOX1‐AS1#1‐transfected cells and thereby sh‐BBOX1‐AS1#1 was chosen to perform the following experiments (Figure 2A). As demonstrated in Figure 2B,C, the proliferation of SiHa and HeLa cells was restrained by knockdown of BBOX1‐AS1. Moreover, decreased expression of BBOX1‐AS1 enhanced the apoptosis capability of SiHa and HeLa cells (Figure 2D). Through Western blot analysis, an increased expression of Bax, cleaved‐caspase 3, cleaved‐caspase 6, cleaved‐caspase 9 and cleaved‐PARP and a decreased expression of Bcl‐2 were observed in SiHa and HeLa cells, indicating that BBOX1‐AS1 depletion could result in strengthened ability of cell apoptosis (Figure 2E). Furthermore, in SiHa and HeLa cells, downregulation of BBOX1‐AS1 suppressed cell migration and invasion (Figure 2F). Similarly, BBOX1‐AS1 deficiency induced a downregulation of MMP2, MMP9, N‐cadherin, Vimentin, Slug, Twist and an upregulation of E‐cadherin, suggesting that cell metastasis was inhibited by BBOX1‐AS1 silence in SiHa and HeLa cells (Figure 2G). More importantly, immunofluorescence detected an enhanced expression of E‐cadherin but a decreased expression of N‐cadherin, verifying that silenced BBOX1‐AS1 exerted suppressive function on epithelial‐mesenchymal transition (EMT) process in SiHa and HeLa cells (Figure 2H). In sum, knockdown of BBOX1‐AS1 represses CC progression.

### 3.3 BBOX1‐AS1 sponges miR‐361‐3p in CC

**FIGURE 3:**
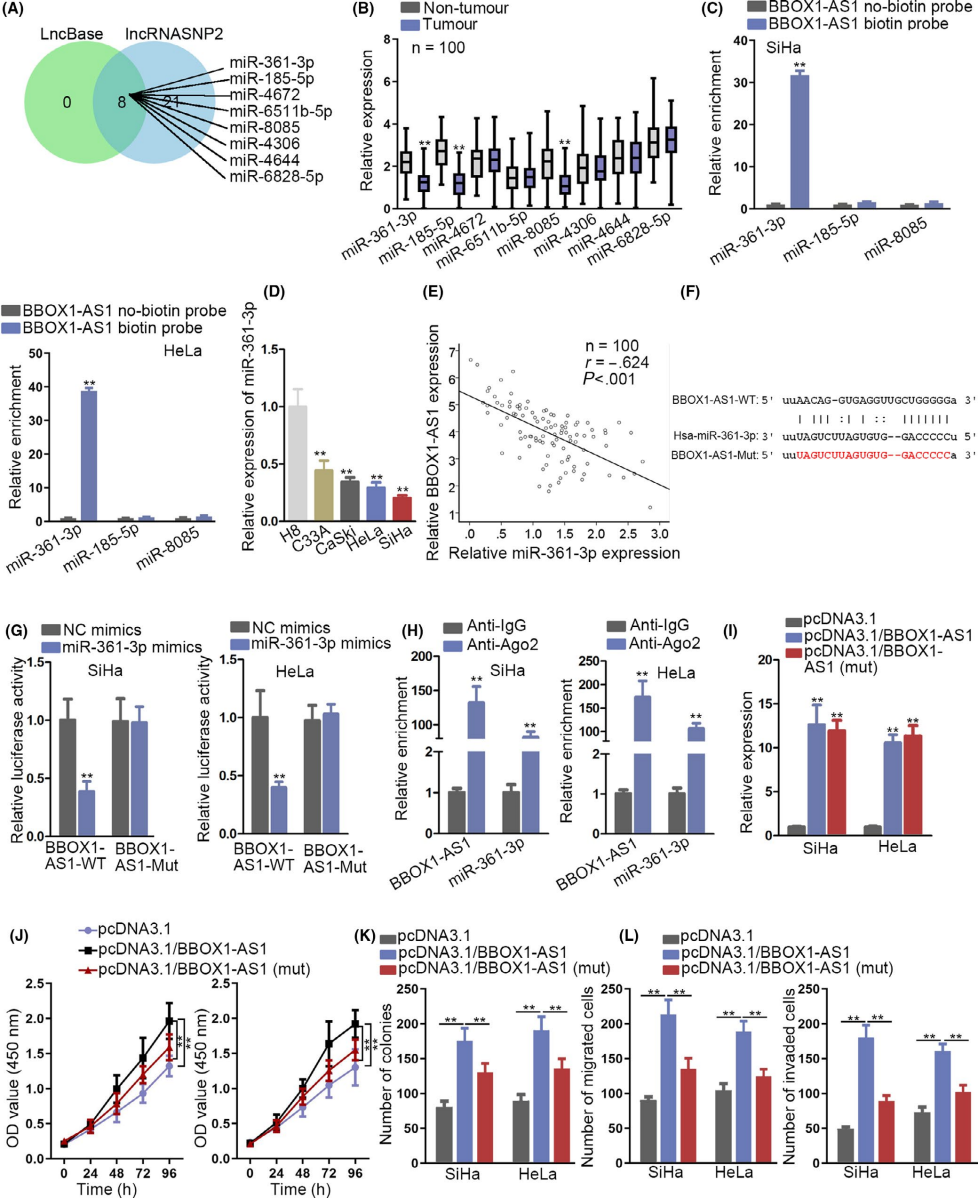
BBOX1 antisense RNA 1 (BBOX1‐AS1) sponges miR‐361‐3p in cervical cancer (CC). A, Target miRNAs of BBOX1‐AS1 were obtained through searching LncBase and lncRNASNP2. B, The expression of these miRNAs was detected in 100 pairs of CC samples and adjacent normal specimens through RT‐qPCR analysis. C, RNA pull‐down assay was conducted to analyse the binding capacity between BBOX1‐AS1 and above miRNAs in SiHa and HeLa cells. D, MiR‐361‐3p expression in CC cell lines and normal cervical epithelial cell line was detected via RT‐qPCR. E, Pearson's correlation analysis analysed the correlation between BBOX1‐AS1 and miR‐361‐3p. F, A binding site between BBOX1‐AS1 and miR‐361‐3p was displayed. G and H, Luciferase reporter and RIP assays testified the interaction between BBOX1‐AS1 and miR‐361‐3p. I, The efficiency of BBOX1‐AS1 and BBOX1‐AS1 (mut) overexpression was analysed through RT‐qPCR. J‐L, CCK‐8, colony formation assay and transwell assays were, respectively, applied to analyse cell proliferation and metastasis in transfected cells. ^**^ *P* < .01

Based on the preceding result of this study that BBOX1‐AS1 was mainly distributed in the cytoplasm of CC cells, we speculated that BBOX1‐AS1 might play a regulatory role in CC by sponging specific miRNA. To prove the speculation, the first thing to do was to screen out the particular miRNA. With the employment of LncBase (http://carolina.imis.athena‐innovation.gr/diana_tools/web/index.php?r=lncbasev2/index‐predicted) as well as lncRNASNP2 (http://bioinfo.life.hust.edu.cn/lncRNASNP#!/), 8 target miRNAs of BBOX1‐AS1 were obtained (Figure 3A). Afterwards, the expression of these miRNAs was detected in 100 paired CC samples and adjacent normal specimens through RT‐qPCR analysis, which revealed that the expression of miR‐185‐5p, miR‐361‐3p and miR‐8085 was distinctly downregulated in CC tissues (Figure 3B). To further screen out the specific miRNA, RNA pull‐down assay was carried out in SiHa and HeLa cells and uncovered a significant enrichment of miR‐361‐3p in BBOX1‐AS1 biotin probe group (Figure 3C). Later on, a remarkably downregulated miR‐361‐3p in CC cell lines was detected via RT‐qPCR (Figure 3D). Moreover, there was a negative correlation between BBOX1‐AS1 expression and miR‐361‐3p expression (Figure 3E). After searching starBase (http://starbase.sysu.edu.cn/), a complementary sequence between BBOX1‐AS1 and miR‐361‐3p was revealed (Figure 3F). Luciferase reporter assay was then applied and depicted a reduced luciferase activity of BBOX1‐AS1‐WT caused by upregulation of miR‐361‐3p in SiHa and HeLa cells. As for the luciferase activity of BBOX1‐AS1‐Mut, no clear change could be observed after upregulating miR‐361‐3p (Figure 3G). Furthermore, in SiHa and HeLa cells, both BBOX1‐AS1 and miR‐361‐3p were enriched in anti‐Ago2 group not in IgG (Figure 3H). To further test whether BBOX1‐AS1 exerts crucial function on CC progression via sponging miR‐361‐3p, we intended to mutate the sequence of BBOX1‐AS1 binding with miR‐361‐3p. Prior to that, a satisfactory efficiency of BBOX1‐AS1 and BBOX1‐AS1 (mut) overexpression was obtained through RT‐qPCR analysis (Figure 3I). Thereafter, results demonstrated that mutation of BBOX1‐AS1 partly reversed the promoting function of BBOX1‐AS1 overexpression on cell proliferation (Figure 3J,K; Figure S1C). Moreover, the facilitating effect induced by overexpression of BBOX1‐AS1 on cell migration and invasion was offset to some extent after BBOX1‐AS1 mutation (Figure 3L; Figure S1D). Taken together, BBOX1‐AS1 sponges miR‐361‐3p in CC.

### 3.4 HOXC6 is testified to be a downstream target of miR‐361‐3p in CC

**FIGURE 4:**
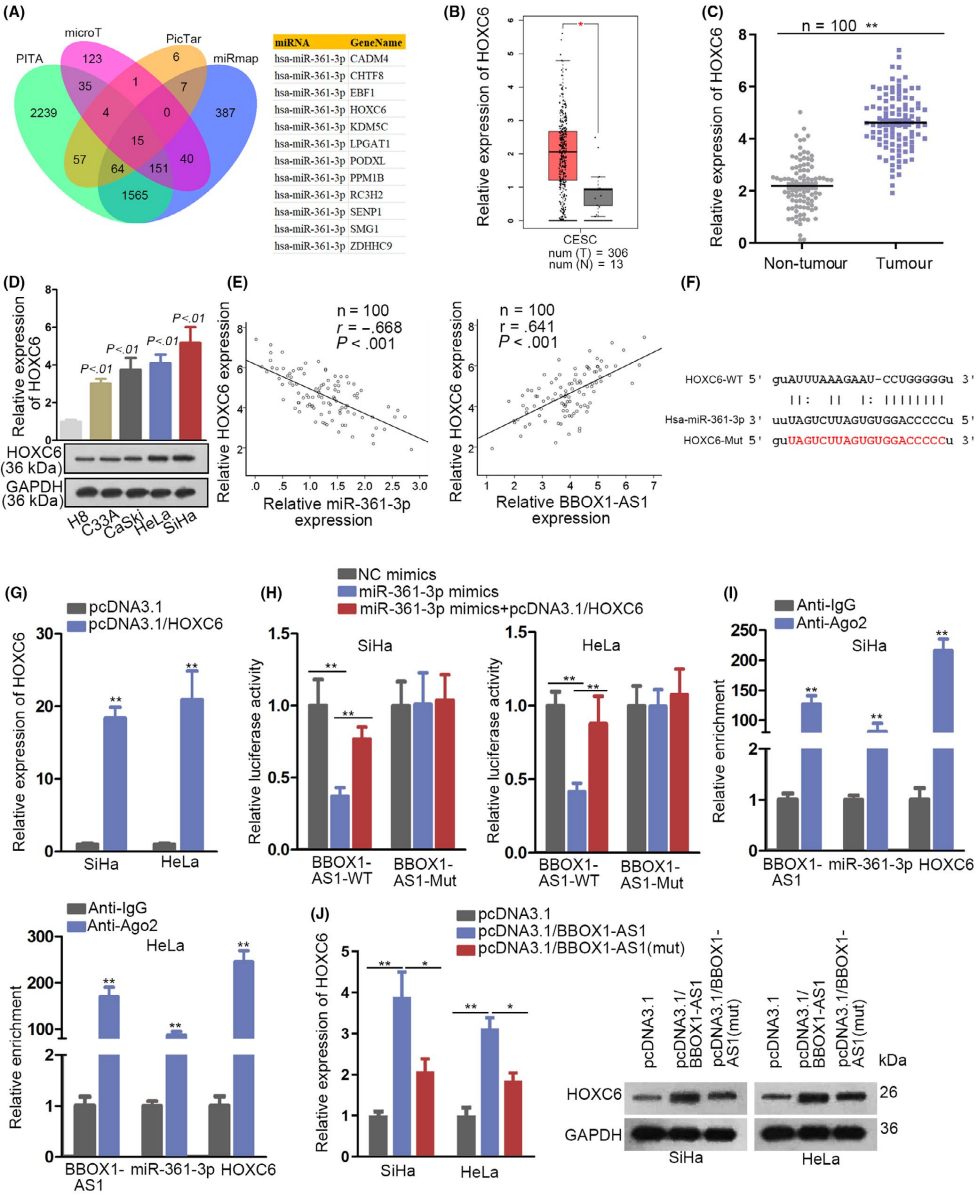
Homeobox C6 (HOXC6) is testified to be a downstream target of miR‐361‐3p in cervical cancer (CC). A, Through utilizing starBase, 12 mRNAs that predicted to have binding capacity with miR‐361‐3p in PITA, miRmap, microT and PicTar databases were screened out. B, The expression level of HOXC6 in CC tissues and neighbouring non‐tumour tissues was obtained from GEPIA. C, RT‐qPCR was used to detect the expression of HOXC6 in CC tissues and neighbouring non‐tumour tissues. D, The expression of HOXC6 in CC cells and H8 cells was detected via RT‐qPCR and Western blot analyses. E, Pearson's correlation analysis revealed the correlation between HOXC6 and miR‐361‐3p (or BBOX1 antisense RNA 1 [BBOX1‐AS1]). F, A binding site between miR‐361‐3p and HOXC6 was predicted via starBase. G, The efficiency of HOXC6 overexpression in SiHa and HeLa cells was examined by RT‐qPCR. H and I, The interaction between RNAs (BBOX1‐AS1, miR‐361‐3p and HOXC6) was confirmed by luciferase reporter and RIP assays. J, The expression of HOXC6 in different groups was analysed via RT‐qPCR. ^*^ *P* < .05, ^**^ *P* < .01

For the purpose of finding the target gene of miR‐361‐3p in CC, we first utilized starBase and screened out 12 mRNAs that predicted to have binding capacity with miR‐361‐3p in PITA, miRmap, microT, miRmap and PicTar databases (Figure 4A). Then, these mRNA expressions were revealed through GEPIA analysis and only HOXC6 expression was found prominently upregulated in CC tissues (Figure 4B, Supplementary Figure 2). Next, HOXC6 expression was higher in CC tissues in comparison with that in neighbouring non‐tumour tissues (Figure 4C). Besides, RT‐qPCR and Western blot analyses detected a significant upregulation of HOXC6 in CC cells compared with that in H8 cells (Figure 4D). More importantly, Pearson's correlation analysis revealed that HOXC6 was negatively correlated with miR‐361‐3p whereas positively correlated with BBOX1‐AS1 (Figure 4E). Afterwards, a binding site between miR‐361‐3p and HOXC6 was predicted via starBase (Figure 4F). Additionally, BBOX1‐AS1 expression was conspicuously increased in SiHa and HeLa cells by transfection with pcDNA3.1/HOXC6 (Figure 4G). To further testify the interaction between RNAs (BBOX1‐AS1, miR‐361‐3p and HOXC6), luciferase reporter and RIP assays were conducted. According to the data from the assays, the lowered luciferase activity of BBOX1‐AS1‐WT induced by miR‐361‐3p upregulation could be rescued by overexpressing HOXC6, while there were no obvious changes concerning the luciferase activity of BBOX1‐AS1‐Mut among different groups (Figure 4H). In addition, BBOX1‐AS1, miR‐361‐3p and HOXC6 were confirmed to exist in RNA‐induced silencing complexes (RISCs) (Figure 4I). Furtherly, an elevated expression of HOXC6 was induced by BBOX1‐AS1 overexpression and then the effect was partly reversed after mutation of BBOX1‐AS1 binding sequence with miR‐361‐3p, which indicated that there might be another mechanism of BBOX1‐AS1 in regulation of HOXC6 expression besides sponging miR‐361‐3p (Figure 4J). All in all, HOXC6 is a downstream target of miR‐361‐3p in CC.

### 3.5 BBOX1‐AS1 promotes the mRNA stability of HOXC6 via HuR in CC

**FIGURE 5:**
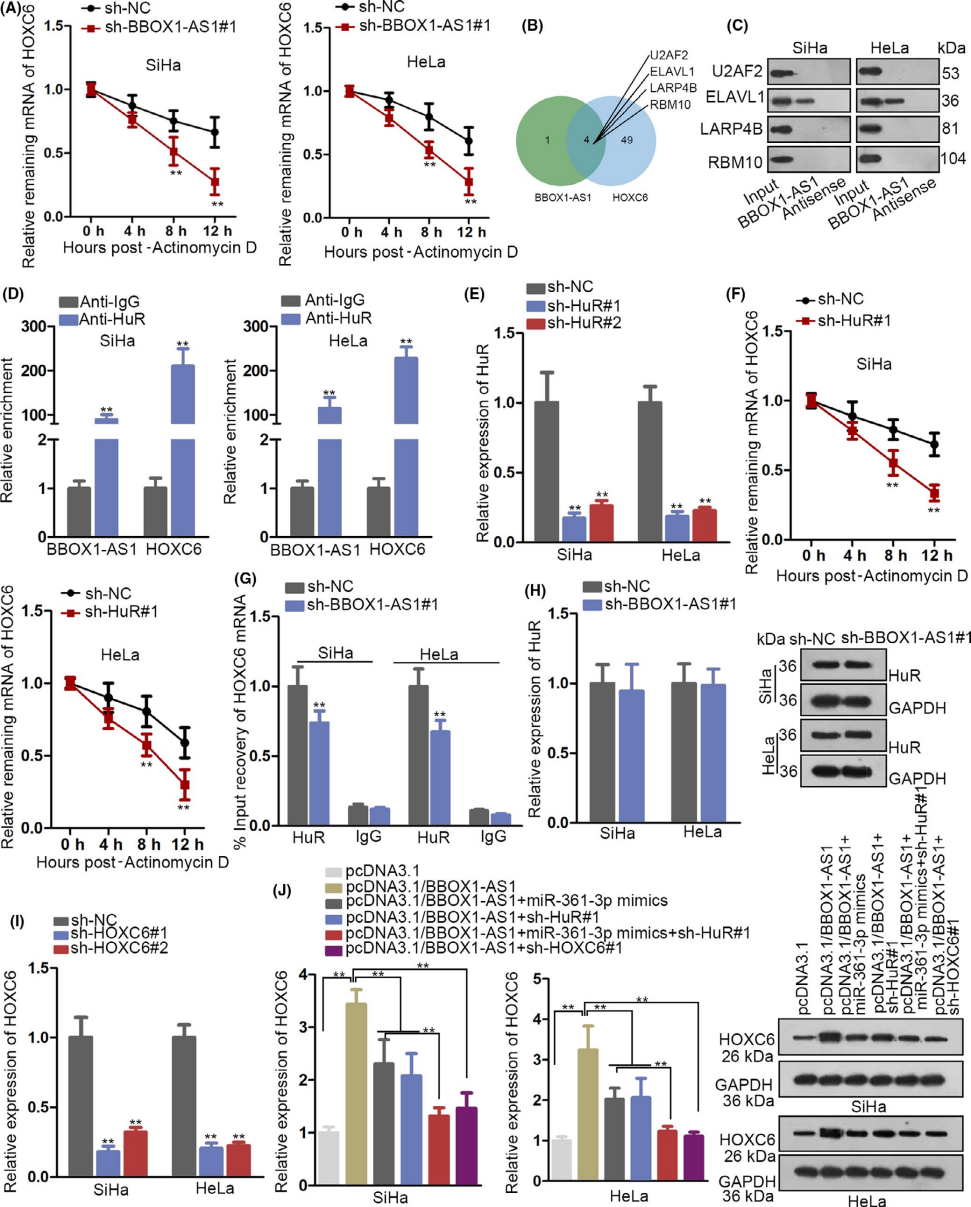
BBOX1 antisense RNA 1 (BBOX1‐AS1) promotes the mRNA stability of homeobox C6 (HOXC6) via HuR in cervical cancer (CC). A, The mRNA expression of HOXC6 was detected by utilizing RT‐qPCR after SiHa and HeLa cells were treated with Actinomycin D. B, Through searching starBase, 4 RBPs (U2AF2, ELAVL1, LARP4B and RBM10) were found to be able to bind with BBOX1‐AS1 (or HOXC6). C, RNA pull‐down assay demonstrated that only ELAVL1 (also known as HuR) bound with BBOX1‐AS1 in SiHa and HeLa cells. D, RIP assay verified that HuR could bind with BBOX1‐AS1 (or HOXC6) in SiHa and HeLa cells. E, Efficiency of indicated shRNAs for HuR silencing was identified through RT‐qPCR in SiHa and HeLa cells. F, Stability of HOXC6 mRNA was assessed in transfected cells treated with Actinomycin D was analysed in several different time points. G, RIP assays were utilized to analyse the changes in the interaction between HOXC6 mRNA and HuR under BBOX1‐AS1 silence. H, Effect of BBOX1‐AS1 inhibition on HuR expression was evaluated by RT‐qPCR and Western blot analyses. I, The efficiency of HOXC6 knockdown was measured by RT‐qPCR. J, The expression of HOXC6 in SiHa and HeLa cells transfected with different plasmids was detected via RT‐qPCR. ^**^ *P* < .01

According to the above findings, we conjectured that BBOX1‐AS1 might regulate HOXC6 expression not only through ceRNA network but also via the interaction with RBPs. In order to verify the conjecture, we detected the mRNA expression of HOXC6 utilizing RT‐qPCR after treatment with Actinomycin D and the result validated that knockdown of BBOX1‐AS1 could inhibit the mRNA stability of HOXC6 (Figure 5A). Subsequently, through searching starBase, 4 RBPs (U2AF2, ELAVL1, LARP4B and RBM10) were found to be able to bind with BBOX1‐AS1 (or HOXC6) (Figure 5B). Later on, RNA pull‐down assay demonstrated that only ELAVL1 (also known as HuR) bound with BBOX1‐AS1 in SiHa and HeLa cells (Figure 5C). Subsequent RIP assay verified that HuR could bind with BBOX1‐AS1 (or HOXC6) in SiHa and HeLa cells (Figure 5D). What's more, to explore whether HuR elicited significant impact on the mRNA stability of HOXC6, we downregulated HuR in SiHa and HeLa cells by transfection with sh‐HuR#1/2, indicating that sh‐HuR#1 presented with better knockdown efficiency of HuR (Figure 5E). After treatment with Actinomycin D, the mRNA stability of HOXC6 was inhibited by HuR deficiency in SiHa and HeLa cells (Figure 5F). Moreover, after BBOX1‐AS1 was silenced in SiHa and HeLa cells, reduced HOXC6 mRNA binding with HuR was observed (Figure 5G), whereas the mRNA and protein expression of HuR demonstrated no evident changes (Figure 5H). With the intention of proofing that BBOX1‐AS1 regulated HOXC6 expression via both ceRNA and RBP mechanism, we first conducted RT‐qPCR assay and obtained a satisfactory efficiency of HOXC6 knockdown (Figure 5I). Then, RT‐qPCR and Western blot analyses revealed that either upregulation of miR‐361‐3p or knockdown of HuR could partly countervail BBOX1‐AS1 upregulation‐mediated promoting function on the expression of HOXC6. More importantly, depletion of HuR after overexpressing miR‐361‐3p could reverse BBOX1‐AS1 upregulation‐mediated effect on HOXC6 expression. Similarly, the function induced by BBOX1‐AS1 upregulation on HOXC6 expression could be restored by HOXC6 depletion (Figure 5J). In other words, BBOX1‐AS1 facilitates the mRNA stability of HOXC6 via HuR in CC.

### 3.6 BBOX1‐AS1 upregulates HOXC6 expression to accelerate CC progression via miR‐361‐3p and HuR

**FIGURE 6:**
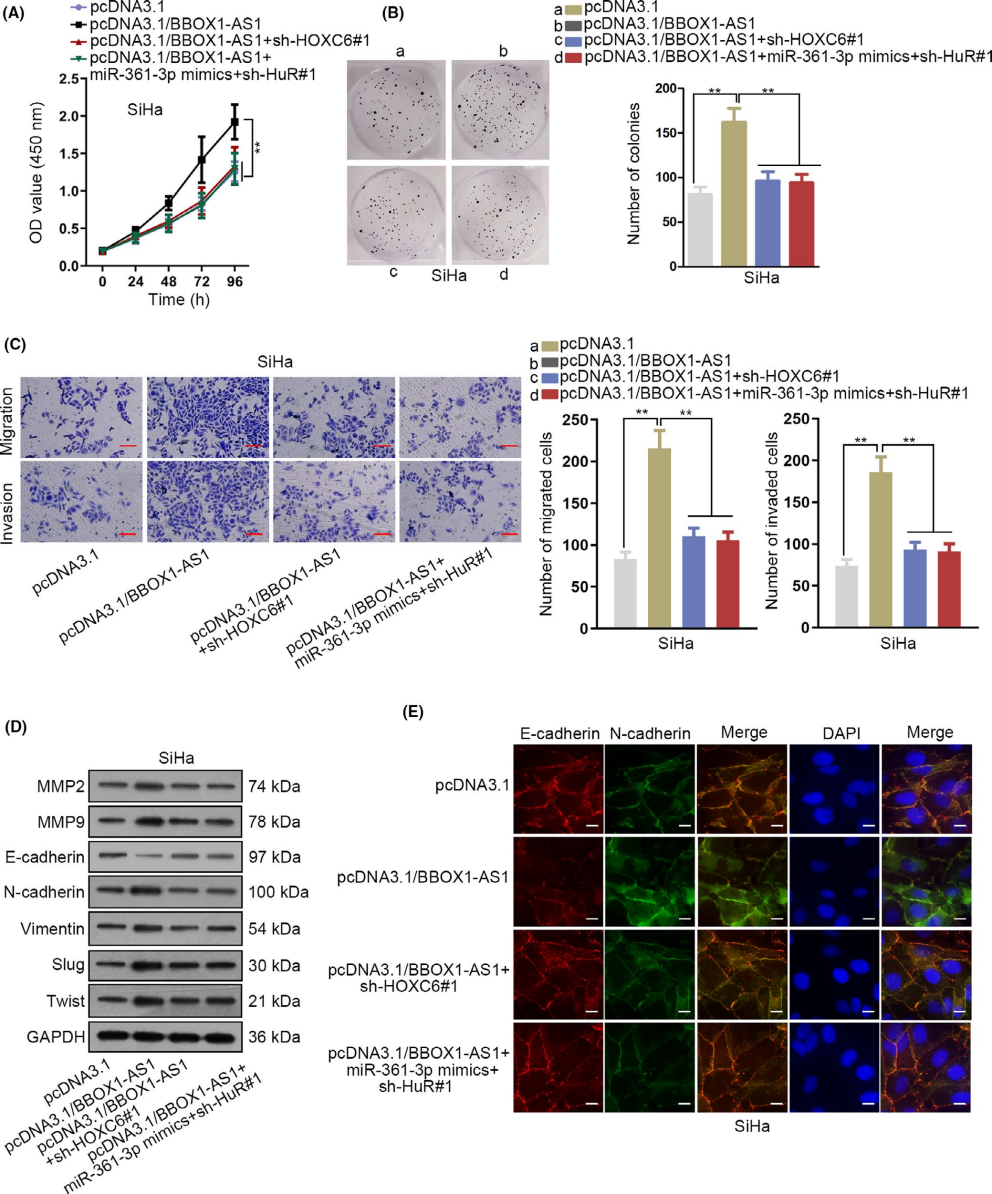
BBOX1 antisense RNA 1 (BBOX1‐AS1) upregulates homeobox C6 (HOXC6) to accelerate cervical cancer (CC) progression via miR‐361‐3p and HuR. A and B, CCK‐8 and colony formation assays were applied to analyse the proliferation ability of transfected cells. C and E, Cell metastasis in transfected cells was assessed by transwell, Western blot and immunofluorescence. ^**^ *P* < .01

To further validate that the above‐mentioned mechanisms of BBOX1‐AS1 could contribute to CC progression, we planned to implement rescue assays. As illustrated in Figure 6A‐B, cell proliferation was facilitated by overexpression of BBOX1‐AS1. But then the effect was reversed by silenced HuR after upregulating miR‐361‐3p; also, the effect could be offset by decreased expression of HOXC6. However, the apoptosis of SiHa cells was hardly affected by BBOX1‐AS1 upregulation or together with other above‐mentioned conditions (data were not shown). This might be explained by initial low apoptotic rate of SiHa cells (nearly 3% as shown in Figure 2D). Moreover, facilitated capability of cell migration and invasion induced by BBOX1‐AS1 upregulation was then reversed by overexpressing miR‐361‐3p together with silencing HuR. And downregulation of HOXC6 could also offset the effect caused by BBOX1‐AS1 upregulation on cell migration and invasion (Figure 6C). In addition, the changes in the metastasis‐associated protein expression in different groups confirmed that HuR depletion on the basis of miR‐361‐3p upregulation could counteract BBOX1‐AS1 upregulation‐induced effect on cell metastasis. And BBOX1‐AS1 upregulation‐induced effect on cell metastasis could also be countervailed by HOXC6 depletion (Figure 6D). Finally, immunofluorescence further validated that not only HuR depletion on the basis of miR‐361‐3p upregulation but also downregulation of HOXC6 could offset BBOX1‐AS1 overexpression‐mediated promoting function on EMT process (Figure 6E). To sum up, BBOX1‐AS1 regulates HOXC6 to modulate CC progression via miR‐361‐3p and HuR.

### 3.7 BBOX1‐AS1 downregulation inhibits the in vivo tumorigenesis of CC

**FIGURE 7:**
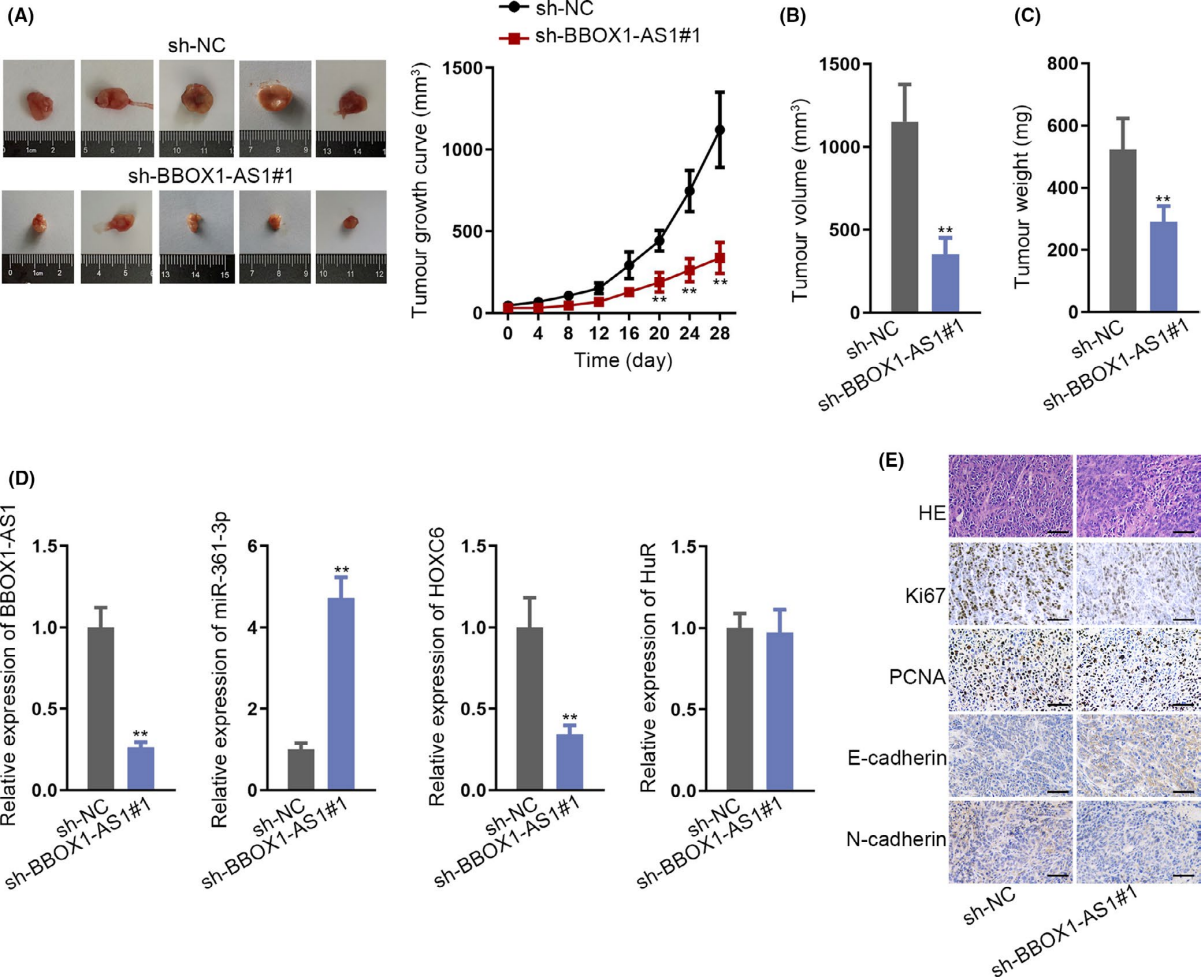
BBOX1 antisense RNA 1 (BBOX1‐AS1) downregulation inhibits the in vivo tumorigenesis of CC. A‐C, Tumour growth, tumour volume and tumour weight were analysed after transfected SiHa cells were injected subcutaneously into nude mice. D, The expression of BBOX1‐AS1, homeobox C6 (HOXC6), miR‐361‐3p and HuR was detected in transfected cells. E, Immunohistochemistry was applied to detect the expression of Ki67, E‐cadherin, PCNA and N‐cadherin in transfected cells. ^**^ *P* < .01

After investigating the key role of BBOX1‐AS1 in vitro tumorigenesis of CC, this part mainly focused on studying the function of BBOX1‐AS1 on the in vivo tumorigenesis of CC. In the first place, SiHa cells transfected with sh‐BBOX1‐AS1#1 or sh‐NC were injected subcutaneously into nude mice. Data from the in vivo assays revealed that knockdown of BBOX1‐AS1 inhibited tumour growth and led to a smaller tumour volume as well (Figure 7A,B). In addition, a lowered tumour weight was observed after silencing BBOX1‐AS1 (Figure 7C). Furthermore, decreased expression of BBOX1‐AS1 gave rise to a reduced expression of BBOX1‐AS1 and HOXC6 whereas an elevated expression of miR‐361‐3p. However, the expression of HuR showed no clear changes after knocking down BBOX1‐AS1 (Figure 7D). More importantly, immunohistochemistry verified that BBOX1‐AS1 deficiency results in a lowered expression of Ki67, PCNA and N‐cadherin whereas an increased expression of E‐cadherin, suggesting that BBOX1‐AS1 depletion could repress cell proliferation and metastasis (Figure 7E). In conclusion, depletion of BBOX1‐AS1 inhibits the in vivo tumorigenesis of CC (Supplementary Figure 3).

## 4 DISCUSSION

Existing literatures have mentioned the involvement of lncRNAs in cancer biology. Known as pivotal regulators, lncRNAs have implicated in the occurrence and development of various human cancer by acting as a ceRNA.^15^, ^16^ Although abundant evidence has highlighted the vital effect of aberrantly expressed lncRNAs in CC progression,^18^, ^19^ the underlying role of BBOX1‐AS1 in CC remains obscure and thus deserves to be studied. In this study, BBOX1‐AS1 expression in CC tissues and cells was significantly upregulated, and upregulated expression of BBOX1‐AS1 predicts poor prognosis of patients with CC. Decreased expression of BBOX1‐AS1 repressed CC cell proliferation and metastasis whereas induced apoptosis.

Growing evidence has indicated that lncRNAs interact with specific miRNA to regulate cancer progression.^20^, ^21^ In the current study, through bioinformatics prediction and molecular mechanism assays, we screened out miR‐361‐3p for further analysis, not only due to its significantly lowered expression in tissues and cells of CC but also its high binding capacity with BBOX1‐AS1 in CC. Though miR‐361‐3p has been unveiled to act as an anti‐tumour gene in cancers,^22^, ^23^ the biological effect that its interaction with BBOX1‐AS1 exerted on CC progression requires to be explored. This study confirmed that miR‐361‐3p bound with BBOX1‐AS1 and negatively correlated with BBOX1‐AS1 in CC. More importantly, functional assays testified that BBOX1‐AS1 regulated CC progression via interaction with miR‐361‐3p.

Homeobox C6 has been confirmed to play a cancerogenic role in some cancers, including CC.^24^, ^25^ However, the interaction of HOXC6 with miR‐361‐3p (or BBOX1‐AS1) in CC cells is still unknown and thus needs exploration. In this study, HOXC6 was confirmed to bind with miR‐361‐3p in CC. Besides, HOXC6 was negatively correlated with miR‐361‐3p but positively correlated with BBOX1‐AS1 in CC. What's more, data in this current study suggested that BBOX1‐AS1 regulated HOXC6 expression not merely by sponging miR‐361‐3p.

ELAV‐like RBP 1, also known as HuR, has been reported to be oncogenic in cancers.^26^, ^27^ Given that HuR, identified as a member of RBPs, has been implicated in the progression of cancers,^28^, ^29^ we speculated that BBOX1‐AS1 might regulate HOXC6 expression via HuR, too. In this study, knockdown of BBOX1‐AS1 was verified to inhibit the mRNA stability of HOXC6 after treatment with Actinomycin D in CC cells. Then, due to bioinformatics and findings from several assays, HuR was validated to bind with BBOX1‐AS1 (or HOXC6) in CC. Moreover, rescues assays confirmed that BBOX1‐AS1 upregulated HOXC6 expression to accelerate CC progression via miR‐361‐3p and HuR. Final in vivo assays further validated the oncogenic effect of BBOX1‐AS1 on the tumorigenesis of CC.

In summary, this study probed into the regulatory function of BBOX1‐AS1 on CC progression. All the data from this research illuminate that BBOX1‐AS1 upregulates HOXC6 expression through miR‐361‐3p and HuR to drive CC progression. This finding provides evidence that BBOX1‐AS1 upregulation contributes to CC progression and predicts poor prognosis of CC patients. Besides, it indicates that BBOX1‐AS1 may be applied as a promising target for the clinical treatment of CC. Nevertheless, the analysis of limited samples is a limitation of our study. Thus, enlarging our sample size and providing more clinical analysis are the research direction in future.

## CONFLICTS OF INTEREST

This study contained no conflicts of interest.

## AUTHORS' CONTRIBUTION

Jun Xu and Baohua Yang designed the article and wrote the manuscript. Lifeng Wang and Yunheng Zhu performed the experiment. Xiuxiang Zhu and Ziyin Xia contributed to the investigation. Zhen Zhao and Ling Xu devoted to the resources. All authors approved the final manuscript.

## Supporting information

Supplementary Material

## Data Availability

Research data are not shared.
