# Supplementary material for: LncRNA BBOX1‐AS1 upregulates HOXC6 expression through miR‐361‐3p and HuR to drive cervical cancer progression
Source: Cell Prolif. 2020 Jun 9;53(7):e12823. doi: 10.1111/cpr.12823 (PMC7377938; doi:10.1111/cpr.12823)
Supplement: Supplementary file 1 — Supplementary Material [file CPR-53-e12823-s001.docx]

**
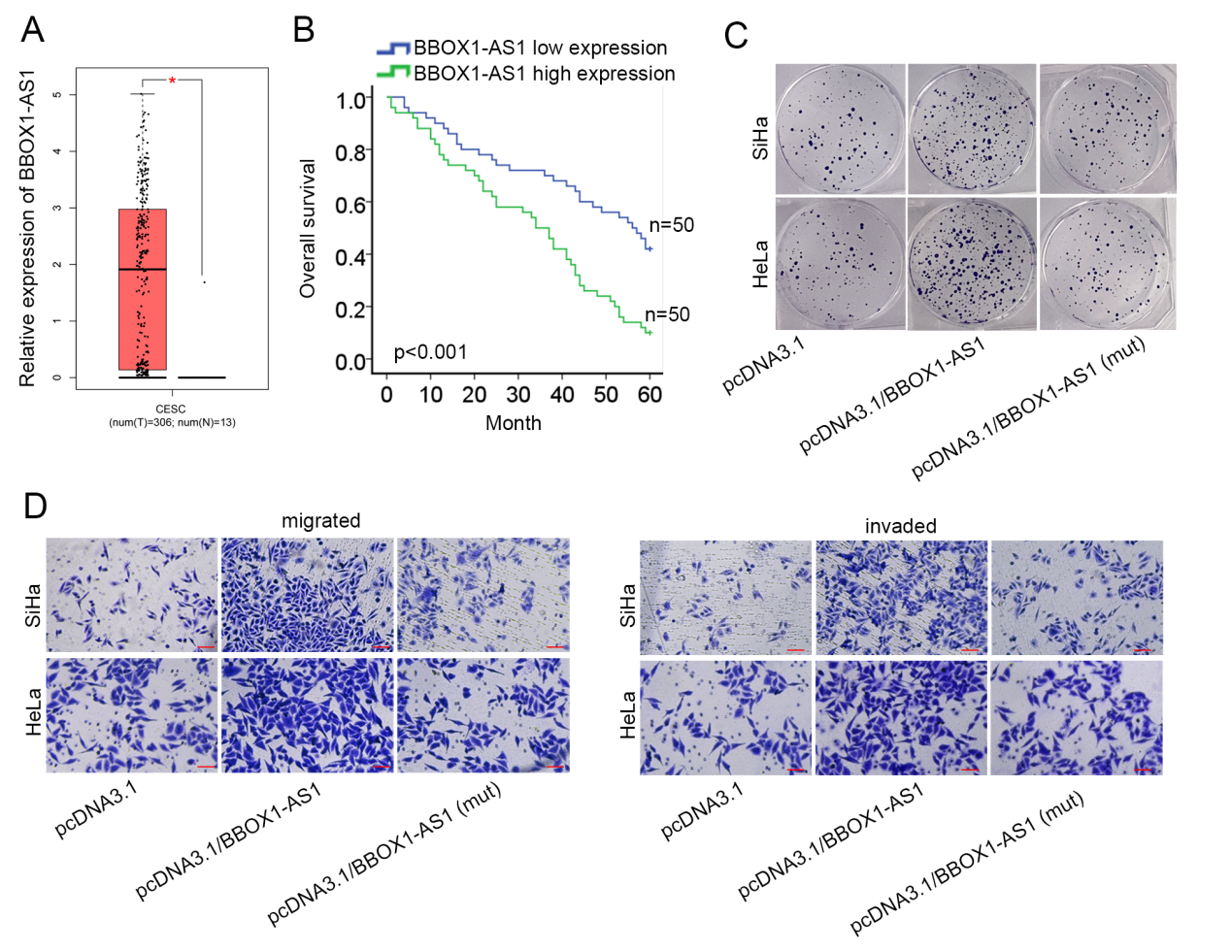
**

**Supplementary Figure 1**

(A) BBOX1-AS1 expression in CC tissues and adjacent normal tissues was uncovered via GEPIA. (B) The overall survival of CC patients with high or low expression of BBOX1-AS1 was analyzed via Kaplan-Meier analysis. (C) The images of colony formation assay conducted in Figure 3K were presented. (D) The images of transwell assay of Figure 3L were displayed. ^*^P < 0.05.


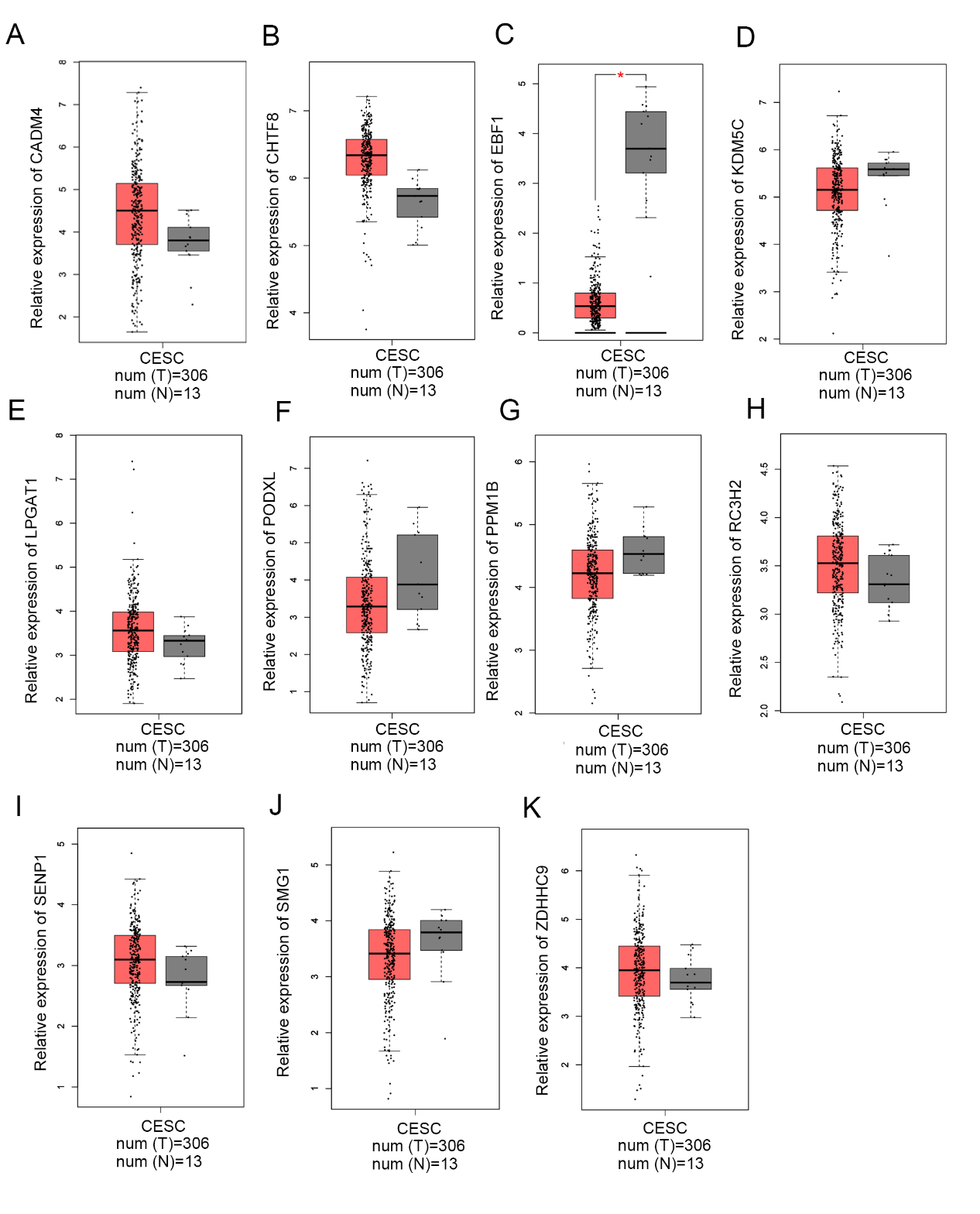


**Supplementary Figure 2**

(A-K) According to GEPIA, the expression of mRNAs in CC tissues and corresponding non-cancer tissues were illustrated. ^*^P < 0.05.

**
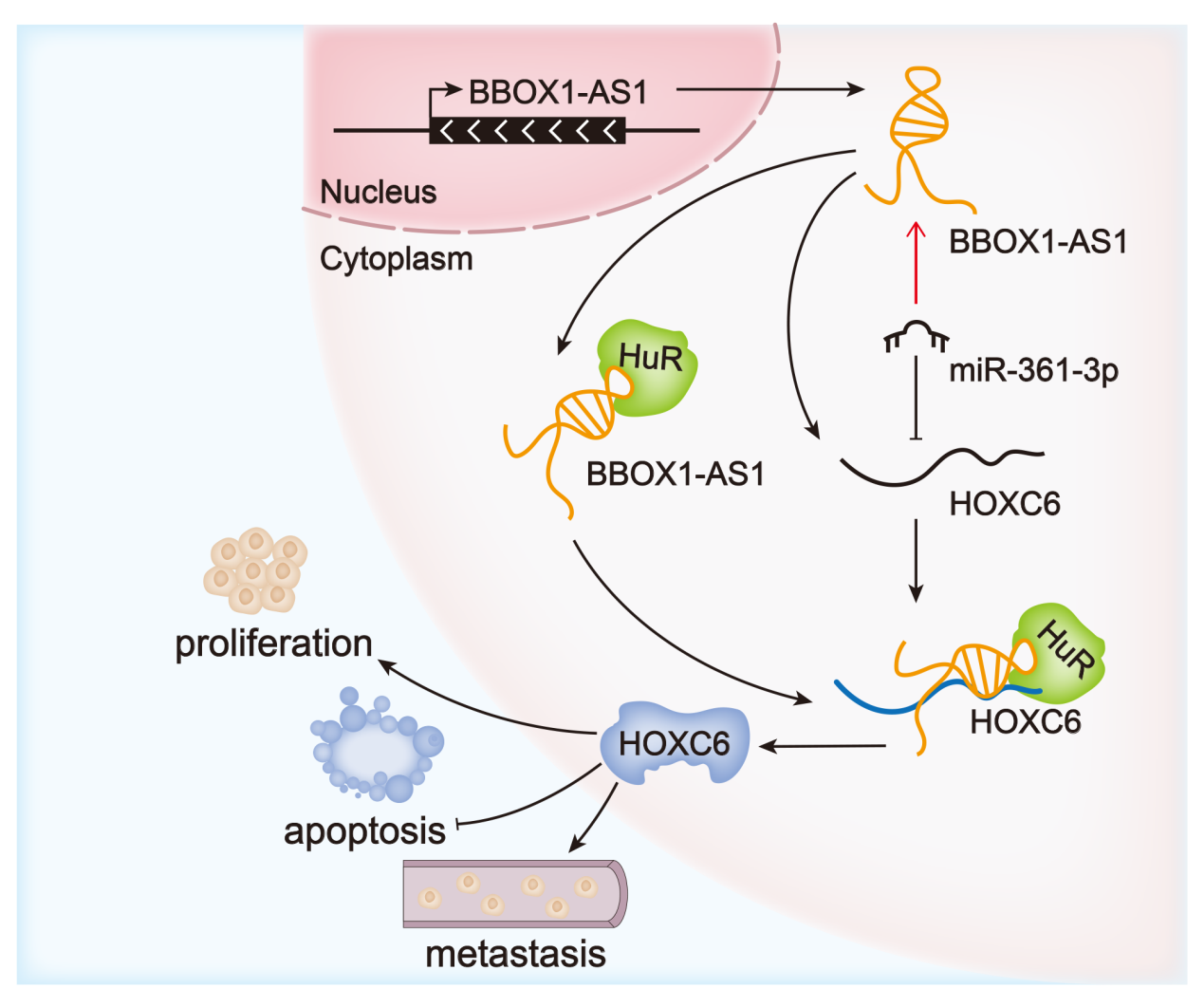
**

**Supplementary Figure 3**

A graphical abstract was utilized to demonstrate the regulatory role of BBOX1-AS1 in CC progression.

**Supplementary Table 1**

47 lncRNAs with high expression in CC tissues were listed.

| AL450992.2 |
| --- |
| AP000251.3 |
| ATP2A1-AS1 |
| BBOX1-AS1 |
| C5orf66-AS1 |
| CDKN2B-AS1 |
| CH17-360D5.2 |
| CH17-360D5.3 |
| CRNDE |
| CTA-384D8.31 |
| CTA-384D8.34 |
| CTA-384D8.35 |
| CTA-384D8.36 |
| CTD-2015H6.3 |
| CTD-2396E7.11 |
| CTD-2510F5.4 |
| CTD-2619J13.13 |
| DGUOK-AS1 |
| FOXD3-AS1 |
| HAGLROS |
| LINC00467 |
| LINC00511 |
| LINC00958 |
| MIR205HG |
| NCK1-AS1 |
| RP11-1149O23.3 |
| RP11-12G12.7 |
| RP11-284F21.10 |
| RP11-295G20.2 |
| RP11-303E16.2 |
| RP11-323C15.2 |
| RP11-357H14.17 |
| RP11-44F14.10 |
| RP11-44F14.2 |
| RP11-452I5.2 |
| RP11-465B22.8 |
| RP11-532F12.5 |
| RP11-545E17.3 |
| RP11-58O9.2 |
| RP11-783K16.5 |
| RP11-7K24.3 |
| RP11-800A3.4 |
| RP3-406A7.7 |
| RP3-512B11.3 |
| RP3-523K23.2 |
| RP6-65G23.3 |
| TYMSOS |

**Supplementary Table 2**

The relationship between BBOX1-AS1 expression and clinical parameters of cervical cancer patients (n=100)

| **Clinical parameters** | BBOX1-AS1 Expression | | P-value |
| --- | --- | --- | --- |
|  | low | high |  |
| **Age** |  |  |  |
| <60 | 20 | 23 | 0.686 |
| ≥60 | 30 | 27 |  |
| **Tumor Size** |  |  |  |
| <4cm | 22 | 9 | 0.009^**^ |
| ≥4cm | 28 | 41 |  |
| **Differentiation** |  |  |  |
| Well | 30 | 11 | <0.001^***^ |
| Poor | 20 | 39 |  |
| **FIGO stage** |  |  |  |
| I-II | 25 | 7 | <0.001^***^ |
| III-IV | 25 | 43 |  |
| **Lymph node metastasis**  No | 28 | 24 | 0.548 |
| Yes | 22 | 26 |  |
| **Distant metastasis** |  |  |  |
| No | 27 | 12 | 0.004^**^ |
| Yes | 23 | 38 |  |

Low/high by the sample median. Pearson χ^2^ test. ^*^P<0.05, ^**^P<0.01, ^***^P<0.001 were considered to be statistically significant.
